# Population Medicine, Population Health, and Population Health Management: Strategies That Meet Society's Health Needs

**DOI:** 10.1016/j.focus.2023.100164

**Published:** 2023-11-08

**Authors:** Irina Arkhipova-Jenkins, Sritha Reddy Rajupet

**Affiliations:** 1 Department of Family, Population and Preventive Medicine, Renaissance School of Medicine, Stony Brook University, Stony Brook, New York; 2 Department of Biomedical Informatics, Renaissance School of Medicine and College of Engineering and Applied Sciences, Stony Brook University, Stony Brook, New York

I knew upon glancing over the chart clipped to the exam room door that it was going to be another challenging clinic visit. With a history of heart disease, hypertension, and poorly controlled diabetes, Mrs. C, an African American woman from Brownsville, one of the poorest New York City neighborhoods, was unfortunately not an uncommon patient. Seeing her hemoglobin A1c jump into the double digits, coupled with uncontrolled blood pressure, heightened my unease.

As I entered the exam room, Mrs. C unsteadily propped herself up on the exam table, coughed hard into her mask, and looked at me with eyes that conveyed a weary sadness. Her presenting complaints did not betray her history: a hardworking 58-year-old mother of four, struggling with the recent passing of her own live-in mother because of COVID-19. Depression became an immediate and necessary part of the visit agenda. Mrs. C's anxiety was palpable as she took breaths while I pressed my stethoscope to her back.

## The Evolution of Population Health

Unfortunately, Mrs. C's story is not unique. It has long been recognized that the majority of health outcomes are influenced by social circumstances, environmental exposures, behavioral patterns, and genetics and <15% come from medical care.^1^ Each person's risk of illness must be considered in the context of disease risk of the population to which he or she belongs, lending truth to the statement that one's ZIP code has greater impact on health than one's genetics. This powerful link between individual and population health outcomes was first articulated in 2003 by Kindig et al. as the concept of *population health*, defined as the health outcomes of a group of individuals, including the distribution of such outcomes within the group.^2^ Kindig conceptualized populations as geographically defined communities or subgroups of people with a shared racial/ethnic background or comorbid conditions.^2^

Population health garnered national attention in 2007 when Berwick et al. at the Institute for Healthcare Improvement included improving the health of populations as a component of the Triple Aim, a three-pronged strategy for transforming the American healthcare system.^3^ Within a healthcare improvement context, the term population was redefined as clinical populations managed by the same healthcare organization or covered under the same health insurance program.^4^^,^^5^

The shift away from managing the needs of individual patients toward broader strategies to improving the health of patient populations created the field of population medicine. According to the Institute for Healthcare Improvement, population medicine facilitates the design, delivery, coordination, and payment of high-quality healthcare services to manage the Triple Aim for a population.^5^^,^^6^ Population medicine is often used interchangeably with the term population health management, as both refer to practices that prioritize population health outcomes for clinical populations compared with the broader general public.^5^^,^^7^ Yet, population medicine and population health management are not synonymous. Population medicine prioritizes direct patient care aspects of population health and calls for a combination of clinical expertise and proficiency in population health strategies to fulfill critical healthcare organizational functions. This includes optimizing preventive health services delivery to address gaps in disease prevention and improving the quality and safety of delivered care. Graduate medical training in preventive medicine uniquely equips preventive medicine physicians with this skill set.^8^ In contrast, population health management is mainly concerned with indirect aspects of medical care, does not require physician-level clinical expertise, and focuses on streamlining care delivery and ensuring financial sustainability of healthcare operations.

**Figure 1:**
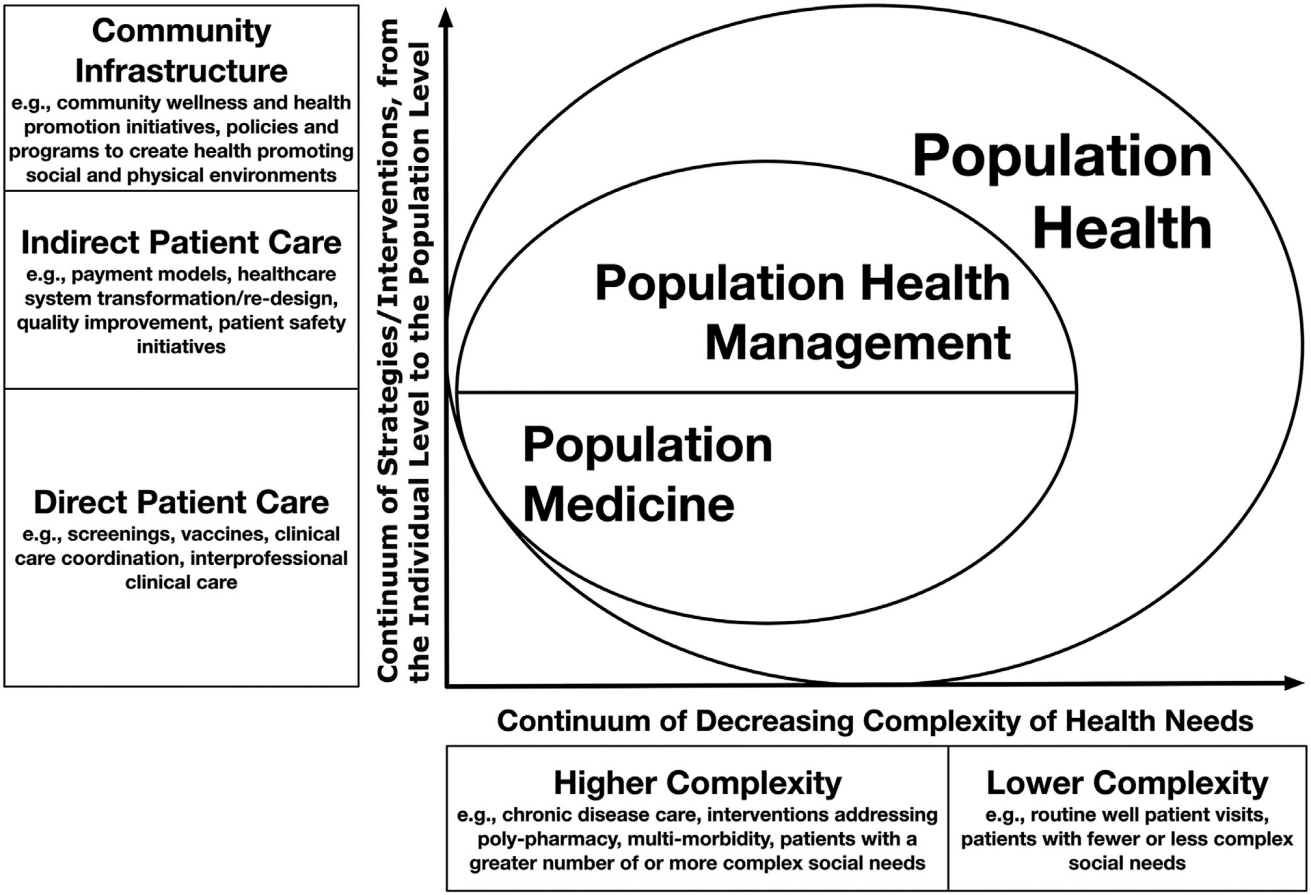
Classification of population health, population medicine, and population health management. *Note*: Figure 1 provides a visual comparison of population health, population medicine, and population health management. The two overlapping concentric shapes in the center of the figure depict the relationship between these three definitions. The largest concentric shape depicts population health, which represents a combination of the clinical population of patients managed by the healthcare organization and the local geographically defined population. The second smaller overlapping concentric shape represents a sum of activities and functions that fall within the domains of population medicine and population health management. As shown by labels on the vertical (Y) axis, these activities and functions cumulatively address a broad spectrum of patient care (both direct and indirect) and health and wellness needs, from the individual to the population levels. The horizontal (X) axis represents a spectrum of complexity of individual health needs ranging from health promotion and wellness and routine preventive care to more complex needs involving chronic disease care and longitudinal management of more complex and resource intensive conditions.

Figure 1 illustrates a complex interplay between different socioeconomic, cultural, environmental, and individual-level factors, cumulatively known as social determinants of health, that influence disease risks within populations and shape health outcomes of people like Mrs. C. A schoolteacher and determined single mother of four, she worked two jobs to scrape together a household income just above the federal poverty line, half of which went to rent. She relied on Medicaid coverage for her chronic conditions and basic preventive services. However, with only one safety net hospital and two community pharmacies in the neighborhood and no reliable access to transportation, she struggled to access timely and effective care to meet her health needs. Sporadic clinic visits and insufficient follow-up could not keep her diabetes, hypertension, and depression under control. Rather than providing longitudinal chronic disease management and addressing health maintenance and prevention needs, her clinicians instead had to prioritize acute care to tackle disease exacerbations.

## Challenges and Opportunities

Grappling with ways to address the social challenges and adverse health effects exemplified by the story of Mrs. C is the essence of population health. The complexity and interconnected nature of these challenges demonstrates that no single model or approach can adequately address both her illness and its underlying causes. Improving population health outcomes requires a multifaceted approach that simultaneously intervenes at the three levels described below. Table 1 provides select examples of the three levels of population health interventions (e.g., care models and strategies) that offer promising results.

**Table 1:** Challenges and Promising Developments in Population Health, Population Medicine, and Population Health Management

Population health needs^a^	Healthcare management strategies^a^	Examples of existing and emerging care delivery models and approaches	Case examples of the three levels of population health strategies
Societal infrastructure (care of community)	Strategies to enhance socioeconomic, cultural, and environmental community conditions including education, employment, recreational opportunities, food security, and access to transportation.	Complex chronic disease care delivery models: ChenMed, Iora Health, Care More, and City Block Condition specific care models: Omada Health, Maven Clinic, and Livongo Urgent care and retail care delivery models: CVS, Walgreens, and CityMD Care coordination delivery models: Bamboo Health, Xealth, and naviHealth Telehealth-based care delivery models: Teladoc, MDLive, and Doctor on Demand Payment models: Pre-payment plans: Medicare Advantage, Medicaid Managed Care, Medicare Shared Savings Program, bundled payments, Accountable Care Organizations, and Clinically Integrated Networks Hybrid payer and health care provider models: Kaiser, Geisinger, Intermountain Healthcare Other: risk-based payment contracts, value-based payments, and capitated payments Emerging technologies and services that can enhance care delivery: healthcare analytics, artificial intelligence, precision medicine, cost transparency, social justice and community activism, and employer-sponsored wellness programs	Level 1: Addressing social determinants of health at the community level Springboard Healthy Scranton Initiative by the Geisinger health system attempted to transform community health care at its core by focusing on preventive care, behavioral health, and supporting local economic growth. Level 2: Ensuring patient-centered and accessible care CityBlock provides a spectrum of patient-centered services ranging from primary and preventive care to addressing housing and food security. Level 3: Building sustainable institutional infrastructure for primary care delivery Bamboo Health uses an innovative healthcare analytic platform to provide real-time patient care input to primary care practices to facilitate care coordination.
Developing institutional infrastructure to support primary care services	Interventions to build interpersonal infrastructure that support improved social life and connectedness to one's community and address social isolation.		
Wellness (care of physical health)	Interventions to enable access to healthy nutrition, physical activity outlets, behavioral interventions to reduce screen time, and programs to support smoking cessation and psychological wellbeing.		
Primary disease prevention (routine clinical care)	Cancer screening, genetic screenings for predispositions to hereditary diseases and genetically driven reactions to pharmacologics, dental care, and preventive immunizations.		
Chronic disease management (secondary clinical care)	Optimizing health while managing chronic conditions (e.g., hypertension, diabetes, heart failure, depression, and others). This category represents the greatest immediate and most proximal opportunity for improving health and reducing avoidable costs.		
Acute care (tertiary and quaternary clinical care)	Surgical interventions, intensive and costly chemotherapeutics, dialysis centers, and other care for patients with complex care needs. The category represents the costliest and the most resource-intensive domain.		

a Categorization of population health needs and some of the recommended health care management strategies were adapted from the book by Neuwirth.^9^

First, healthcare systems must focus their efforts beyond their clinical populations to prioritize upstream strategies to address social determinants that impact the health of their communities.^10^ This means working collaboratively with partners from other sectors of society, such as local public health departments, community organizations, and government, to build a health-promoting social infrastructure that addresses housing, education, neighborhood safety, transportation, and food security. One example of such an effort is the Springboard Healthy Scranton Initiative implemented by the Geisinger health system to address community health needs in Scranton, Pennsylvania.^11^ Geisinger's effort included initiatives such as fresh food pharmacy to address food insecurity and expanded access to addiction medicine services for substance use disorders.

Second, health systems need to utilize population health approaches to provide patient-centered, accessible, and efficient health care. This includes effective patient segmentation according to medical and social needs to identify the types and complexity of needed health surveillance, chronic disease care, and patterns of health outcomes that indicate the need to explore social determinants to tailor the delivery of clinical, social, and community-based interventions.^12^ Some tools and approaches that have shown promise include remote monitoring, digital health technology, integration of home-based care, and developing multidisciplinary care teams able to engage with the community to address community health needs. To be successful, these population health strategies require health system leadership's alignment with the goals and approaches to population health efforts, implementing more sophisticated methods of healthcare financing that integrate new forms of payment with mechanisms of population health measurement, as well as mechanisms for continuous care quality improvement. CityBlock, based in New York City, attempts to tackle this complex task by delivering a spectrum of patient-centered services ranging from primary and preventive care to addressing housing and food insecurities.^13^

Third, health systems need to enable an effective and sustainable primary care infrastructure which provides adequate resources and support for primary care clinicians. For example, Boston-based healthcare technology solutions company Bamboo Health aims to assist primary care coordination by offering a novel health analytics platform that provides real-time patient care data to primary care teams.^14^

## Future Directions

The fields of population health, population medicine, and population health management are continuously evolving. Although existing models and strategies have their limitations, they each can offer unique solutions to address different facets of population health. Achieving meaningful progress in improving health outcomes for all Americans, including those experiencing immense difficulties like Mrs. C, requires grappling with existing challenges while embracing innovation and a collaborative approach to address each of the three levels of population health needs.

## CRediT authorship contribution statement

**Irina Arkhipova-Jenkins:** Conceptualization, Methodology, Formal analysis, Visualization, Writing – original draft, Writing – review & editing. **Sritha Reddy Rajupet:** Conceptualization, Methodology, Formal analysis, Writing – original draft, Writing – review & editing.
